# Positive Fecal Occult Blood Test and Colonoscopy With Histopathology Findings in Saudi Adults

**DOI:** 10.7759/cureus.43312

**Published:** 2023-08-10

**Authors:** Waleed M Alhuzaim, Ghaida A Alloqmany, Norah I Almedemgh, We’am Aldaham, Somayah Alkhenaizan, Shahad Hadal

**Affiliations:** 1 Department of Medicine, Imam Mohammed bin Saud Islamic University, Riyadh, SAU; 2 College of Medicine, Imam Mohammed Bin Saud Islamic University, Riyadh, SAU; 3 College of Medicine, Imam Mohammed bin Saud Islamic University, Riyadh, SAU

**Keywords:** gi symptoms variable, predictor, histopathology, colonoscopy/sigmoidoscopy, fobt, colorectal cancer

## Abstract

Background/aims

Colorectal cancer cases are on the rise in developing countries, necessitating dependable detection tests. Moreover, medical procedures have become increasingly burdensome for both patients and healthcare professionals. This study aims to delve deeper into the fecal occult blood test (FOBT) as a potential solution.

Settings and design

The research took place at the Gastroenterology Specialized Clinic (Human Clinic) in Riyadh, Saudi Arabia. The study gathered results from colonoscopy/sigmoidoscopy, histopathology, and FOBT screening. Essential variables linked to colorectal cancer, including gastrointestinal symptoms, chronic diseases, hemoglobin (Hgb), body mass index (BMI), and medication usage, were documented to evaluate their potential predictive significance for positive outcomes.

Methods and materials

In line with the study aims, inclusion criteria covered Saudi adults aged 18 and above, experiencing lower GI symptoms, with colonoscopy or sigmoidoscopy, FOBT, and histopathological results. The resultant sample size was 72 patients. Non-Saudi individuals, symptoms-free patients, and those below 18 years were excluded. This retrospective analysis spanned from September 2021 to September 2022. For statistical analysis, after the data rationality was checked, parametric or non-parametric tests were used. P ≤ 0.05 was set as the significant level.

Results

Among the 72 patients, ranging in age from 18 to 80 (mean 42.94), males (57) outnumbered females (15). The average BMI was 27.56, with one-third of patients classified as overweight or obese. A majority of 47 (65.2%) exhibited normal Hgb levels while only five (7%) had abnormal levels. Results from colonoscopy or sigmoidoscopy and FOBT displayed statistical similarity between positive and negative outcomes. Additionally, the notable prevalence of positive results compared to negative ones underscores the resemblance between FOBT and colonoscopy/sigmoidoscopy findings.

Conclusion

Chronic illness, constitutional symptoms, BMI, and Hgb did not display a significant predictive value, However, the group with GI symptoms exhibited a strong prediction for favorable colonoscopy/sigmoidoscopy and histology outcomes. Additional research is necessary to validate these observed patterns.

## Introduction

Background and rationale

Colorectal cancer management is even more complex due to the variations in geography, screening, available treatment, and socioeconomic circumstances of patients. Cases are rising in developing countries, and the goal of devising early detection tests and procedures has become a major effort in the medical community [1].

While more and more people are being tested, The U.S. Preventive Services Task Force recommends colorectal cancer screening for adults 45-75 years of age [2,3].

The Centers for Disease Control and Prevention lists current options for colorectal cancer screening. There are three stool tests available: the guaiac-based fecal occult blood test, the fecal immunochemical test (FIT), which uses antibodies as a blood presence detector, and the FIT-DNA test, which analyzes the DNA in the stool and determines any alteration that may indicate an abnormality [2].

Device-based screenings consist of flexible sigmoidoscopy, colonoscopy, CT colonography (virtual colonoscopy), and sigmoidoscopy. Colonoscopy is a similar test to sigmoidoscopy, but the tube travels the full length of the rectum and colon. CT colonography, or virtual colonoscopy, is less physically intrusive, as it uses X-rays and computer imaging to visually map the entire colon. Currently, it is the gold standard of colorectal cancer screening [4].

Methods based on blood markers are yet to establish their definitive role in the future of colorectal cancer screening. Factors like cost may very well be a major consideration, as it can increase compliance, and there is evidence that also suggests that patients tend to welcome a blood test more than the usual screening procedures, which may be cumbersome, invasive, and generally more expensive [5]. While colonoscopy is considered the superior method, a pros-and-cons analysis remains a factor in many decisions regarding the choice of screening procedure. FOBT has the advantage of having no preparatory requirements, being inexpensive, being home-based, and having no direct risk to the colon [6].

A systematic review of the US Preventive Task Force has concluded that in the aspect of preventing cancer mortality in colorectal cancer, each has varied levels of detection as well as risk and harm [7].

The Center for Disease Control Colorectal Control Program (CRCCP) was evaluated in a study focusing on a cost analysis of screening activities and reports that during the first three years of the control program, the choice between FOBT/FIT and colonoscopy will be critical in terms of timeliness and availability of screening procedures, as more subjects will be tested earlier and quicker at a lower cost. The study recommends that these clinical and non-clinical costs should be a key consideration in the implementation of the program [8].

This study was conducted to explore the predictive value of FOBT for colorectal cancer when compared with screening modalities of colonoscopy/sigmoidoscopy and histopathology among symptomatic adult Saudi patients. Other key variables were tested for association with positive results that could help predict positive colonoscopy/sigmoidoscopy and histopathology results.

## Materials and methods

Ethical approval

This study has been approved by the Institutional Review Board (IRB) of Al-Imam Mohammed bin Saud Islamic University in Riyadh, Saudi Arabia.

Study design

This is a retrospective cohort study covering data from September 2021 to September 2022 at the Human Clinic, Gastroenterology Specialized Clinic in Riyadh, Saudi Arabia. As per the study aims, the inclusion criteria encompass Saudi adults aged 18 and above who have experienced lower GI symptoms, have undergone either colonoscopy or sigmoidoscopy along with FOBT, and possess histopathological evaluation results. Consequently, the resulting sample size is 72 patients. Excluded from the study were non-Saudi patients, as well as those without symptoms, and patients below 18 years of age. Continuous data were meticulously assessed for logical consistency, and means were juxtaposed employing appropriate parametric or non-parametric tests. The utmost confidentiality of the collected data has been scrupulously maintained. The significance test was conducted with P ≤ 0.05 set as the significant level.

The overarching objective of this retrospective cohort study is to discern disparities in histopathological, FOBT, and colonoscopy findings among symptomatic adults.

Data collection method

Patient data were gathered from both paper files and electronic medical documentation at the Human Clinic, Gastroenterology Specialized Clinic in Riyadh, This encompassed comprehensive general and medical information such as age, gender, body mass index (BMI), hemoglobin levels, chronic diseases, medication usage, as well as gastrointestinal and constitutional symptoms. Moreover, we procured results from the FOBT, colonoscopy, sigmoidoscopy, and histopathology analysis. Notably, all participants granted informed consent.

Statistics

This study was analyzed utilizing IBM SPSS version 23 (IBM Corp., Armonk, N.Y.). To delineate the characteristics of study variables, a simple descriptive statistic was employed, utilizing counts and percentages for the categorical and nominal variables while continuous variables were presented through means and standard deviations. A chi-square test was employed to ascertain relationships among categorical variables, with these tests conducted assuming a normal distribution. Binary outcomes were designated as dependent study variables. Employing a binary logistic regression model (BLRM), with backward conditional elimination with enter criteria=0.05 and elimination=0.10, was used to determine the significant predictors of each dependent study variable by 95% confidence intervals. Lastly, a conventional p-value <0.05 was used to reject the null hypothesis.

## Results

Patient demographics

The ages of the subjects ranged from 18 to 80 years, with an average of 42.94. The mean BMI was 27.56. The mean Hgb level was 13.8. The male/female gender distribution was 57 (79.2%) and 15 (20.8%), respectively. As per BMI scores, 25 were overweight and 23 were obese [9]. Normal and underweight patients were 16 and five, respectively. FOBT found 45 positives and 27 negatives. Forty-seven (65.3%) had a normal Hgb count, five were abnormal, and the rest had no data. Thirty of the patients were using medications, and 42 are not. Of those suffering from chronic diseases, 19 were suffering from them and 53 were not (Tables 1, 2).

**Table 1:** Patient demographics BMI: body mass index; Hgb: hemoglobin

Demographics	N	Min	Max	Mean	SD
Age	72	18	80	42.94	12.9
BMI	69	13.10	44.50	27.56	6.3
Hgb	52	8.70	16.40	13.38	1.5

**Table 2:** Characteristics of the 72 study samples FOBT: fecal occult blood test

		Count	%
Total		72	100.0
Gender	Male	57	79.2
	Female	15	20.8
BMI	Underweight	5	6.9
	Normal	16	22.2
	Overweight	25	34.7
	Obese	23	31.9
	N/A	3	4.2
FOBT (+/-)	Negative	27	37.5
	Positive	45	62.5
Hgb	Normal (12-18)	47	65.3
	Abnormal	5	6.9
	N/A	20	27.8
Old Medication	Yes	30	41.7
	None	42	58.3
Chronic Diseases	Yes	19	26.4
	No	53	73.6

Symptoms and chronic diseases

The most prevalent symptom among patients was GI symptoms, comprising 68 (94.4%) of the cases. Constitutional symptoms accounted for six (8.3%), respiratory one (1.4%), and others seven (9.7%). Diabetes (DM) and hypertension (HTN) were equally prevalent as the most common chronic diseases present in seven (9.7%) of all cases. Other conditions were asthma, three (4.2%), post-surgery, heart disease, and digestive, each with two (2.8%). Lipid disease was reported in one (1.4%) case (Table 3).

**Table 3:** Symptoms and chronic diseases GI: gastrointestinal; DM: diabetes mellitus; HTN: hypertension

Variables		Count	%
Total		72	100.0
Symptoms	GI symptoms	68	94.4
	Constitutional symptoms	6	8.3
	Respiratory tract symptoms	1	1.4
	Other	7	9.7
	Condition, not a symptom	1	1.4
Chronic Diseases	DM	7	9.7
	HTN	7	9.7
	Asthma	3	4.2
	Post-surgery	2	2.8
	Lipid disease	1	1.4
	Heart disease	2	2.8
	Digestive system-related conditions	2	2.8
	NA	53	73.6

Colonoscopy and histopathology

Of the 72 participants, 53 (73.6%) underwent colonoscopy. Histopathology examination was done on the same number of cases (Table 4).

**Table 4:** Methods variables

Variables		Count	%
Colonoscopy/Sigmoidoscopy Results	Positive	53	73.6
	Normal	19	26.4
Histopathology	With histopathology	53	73.6
	No pathology	19	26.4

Colonoscopy and FOBT

**Figure 1:**
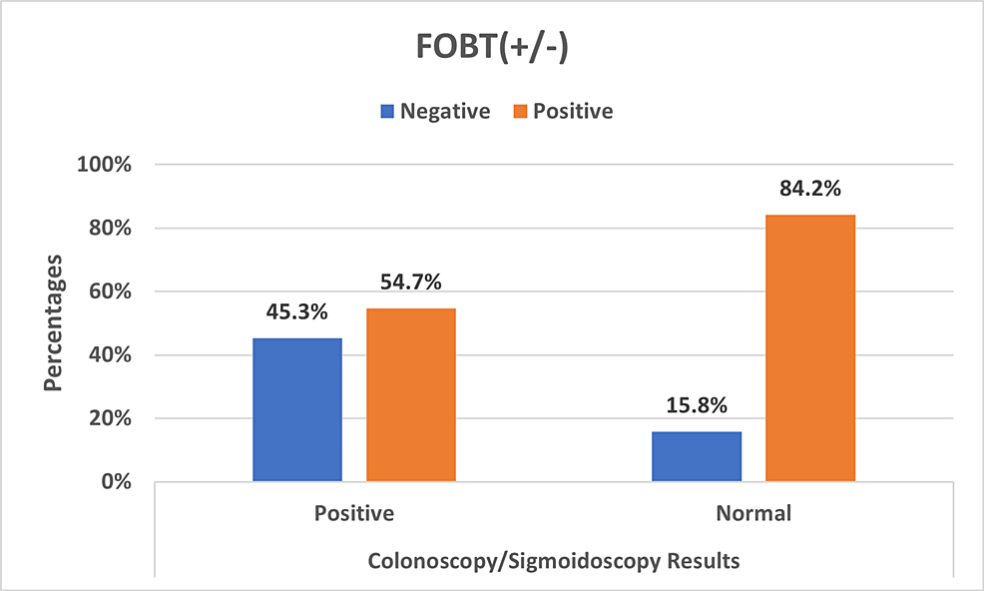
FOBT positive/negative distribution across positive/normal colonoscopy/sigmoidoscopy results FOBT: fecal occult blood test

Figure 1 displays the findings of FOBT across the colonoscopy/sigmoidoscopy results. The FOBT columns provide negative and positive breakdowns of the positive and negative results of colonoscopy. The FOBT negative and positive distributions were tested for the statistical significance of their differences. The p-value was 0.023, indicating that the apparent higher positive results of FOBT are statistically significant, implying that FOBT will generally detect more positives than negatives. This, however, only covers the relationship between the FOBT's negative and positive results.

To examine the relationship between colonoscopy and FOBT results, the positive/negative distributions of colonoscopy and FOBT results were set side by side. The difference was not significant, which implies that there is a basis to claim that the ratio of positive to negative results of FOBT to colonoscopy is not significant.

Histopathology and FOBT

**Figure 2:**
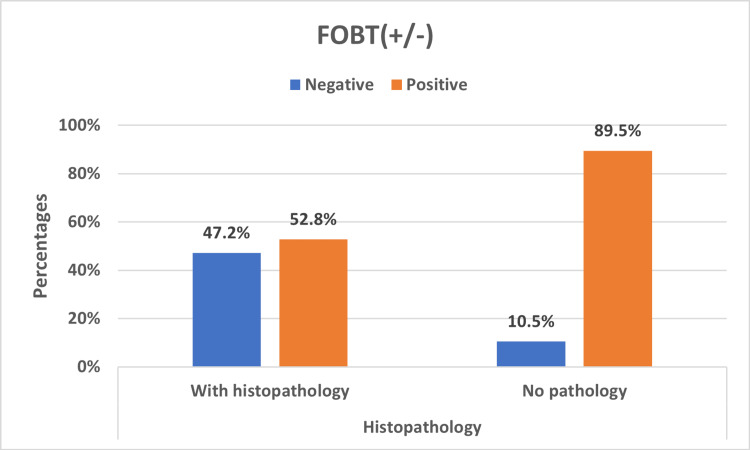
FOBT positive/negative distribution across with and no histopathology groups FOBT: fecal occult blood test

In Figure 2, the FOBT negative and positive distributions were tested for the statistical significance of their differences. The p-value was 0.005, indicating that FOBT will tend to detect more positives than in subjects that had histopathology.

Binary logistic regression analysis

Binary logistic regression analysis was done to determine the contribution of variables to predicting results. Of the variables, histopathology and chronic disease were used in the equation, but only histopathology reached a significance level at the last step (Table 5).

**Table 5:** Binary logistic regression equation variables and results a - variable(s) entered on step 1: colonoscopy/sigmoidoscopy results, histopathology, chronic disease. b - significant using the binary logistic regression model, with backward conditional elimination with enter criteria=0.05, elimination=0.10

Variables in the Equation		B	S.E.	OR	95% C.I. for OR		p-value
					Lower	Upper	
First Step ^a^	Colonoscopy/Sigmoidoscopy Results (Positive)	-0.879	0.766	0.415	0.093	1.865	0.251
	Histopathology (With Histopathology)	-1.545	0.853	0.213	0.040	1.135	0.070
	Chronic Disease (Yes)	1.315	0.721	3.726	0.907	15.304	0.068
	Constant	2.175	0.892	8.804			0.015 ^b^
Last Step ^a^	Histopathology (With Histopathology)	-1.877	0.810	0.153	0.031	0.748	0.020 ^b^
	Chronic Disease (Yes)	1.287	0.714	3.621	0.893	14.681	0.072
	Constant	1.747	0.771	5.738			0.024 ^b^

Key variables in FOBT results

Variables were identified to measure which had a statistically significant relationship with FOBT results (Table 6). Among these were gender, BMI, Hgb, use of medication, chronic disease GI symptoms, and constitutional symptoms. The only variable that tested significantly in predicting positive results was the presence of chronic disease in patients, a finding that deviates from the predictor observed in colonoscopy/sigmoidoscopy and histopathology groups.

**Table 6:** FOBT results across variables FOBT: fecal occult blood test

Variables		Total	FOBT(+/-)		p-value
			Negative	Positive	
Total		72	27 (37.5%)	45 (62.5%)	-
Gender	Male	57	23 (40.4%)	34 (59.6%)	0.33
	Female	15	4 (26.7%)	11 (73.3%)	
BMI	Underweight	5	3 (60.0%)	2 (40.0%)	0.294
	Normal	16	8 (50.0%)	8 (50.0%)	
	Overweight	25	7 (28.0%)	18 (72.0%)	
	Obese	23	9 (39.1%)	14 (60.9%)	
	N/A	3	0 (0.0%)	3 (100.0%)	
Hgb	Normal(12-18)	47	17 (36.2%)	30 (63.8%)	0.95
	Abnormal	5	2 (40.0%)	3 (60.0%)	
	N/A	20	8 (40.0%)	12 (60.0%)	
Use of Medication	Yes	30	9 (30.0%)	21 (70.0%)	0.267
	None	42	18 (42.9%)	24 (57.1%)	
Chronic Disease	Yes	19	3 (15.8%)	16 (84.2%)	0.023
	No	53	24 (45.3%)	29 (54.7%)	
GI Symptoms	Yes	68	26 (38.2%)	42 (61.8%)	0.595
	No	4	1 (25.0%)	3 (75.0%)	
Constitutional symptoms	Yes	6	2 (33.3%)	4 (66.7%)	0.826
	No	66	25 (37.9%)	41 (62.1%)	
Other	Yes	7	4 (57.1%)	3 (42.9%)	0.259
	No	65	23 (35.4%)	42 (64.6%)	

Key variables in colonoscopy/sigmoidoscopy results

The same variables, gender, BMI, Hgb, medication, chronic disease, GI symptoms, and constitutional symptoms, were tested for statistical significance in their relationship with colonoscopy/sigmoidoscopy results (Table 7). The only variable that tested significantly was GI symptoms suggesting that the presence of these symptoms is predictive of positive colonoscopy/sigmoidoscopy results making the variable a significant predictor of the positive results.

**Table 7:** Colonoscopy/sigmoidoscopy results across variables

Variables		Total	Colonoscopy/Sigmoidoscopy Results		p-value
			Positive	Normal	
Total		72	53 (73.6%)	19 (26.4%)	-
Gender	Male	57	44 (77.2%)	13 (22.8%)	0.179
	Female	15	9 (60.0%)	6 (40.0%)	
BMI	Underweight	5	4 (80.0%)	1 (20.0%)	0.843
	Normal	16	11 (68.8%)	5 (31.3%)	
	Overweight	25	18 (72.0%)	7 (28.0%)	
	Obese	23	17 (73.9%)	6 (26.1%)	
	N/A	3	3 (100.0%)	0 (0.0%)	
Hgb	Normal (12-18)	47	36 (76.6%)	11 (23.4%)	0.661
	Abnormal	5	3 (60.0%)	2 (40.0%)	
	N/A	20	14 (70.0%)	6 (30.0%)	
Old Medication	Yes	30	20 (66.7%)	10 (33.3%)	0.258
	None	42	33 (78.6%)	9 (21.4%)	
Chronic Disease	Yes	19	12 (63.2%)	7 (36.8%)	0.288
	No	53	41 (77.4%)	12 (22.6%)	
GI Symptoms	Yes	68	53 (77.9%)	15 (22.1%)	0.001
	No	4	0 (0.0%)	4 (100.0%)	
Constitutional Symptoms	Yes	6	3 (50.0%)	3 (50.0%)	0.171
	No	66	50 (75.8%)	16 (24.2%)	
Other	Yes	7	5 (71.4%)	2 (28.6%)	0.890
	No	65	48 (73.8%)	17 (26.2%)	

The same above variables were tested for their individual relationship with histopathology results (Table 8). Again, only the presence of GI symptoms significantly predicted a positive histopathology result.

**Table 8:** Individual relationship of variables with histopathology results BMI: body mass index; Hgb: hemoglobin

Variables		Total	Histopathology		p-value
			With histopathology	No pathology	
Total		72	53 (73.6%)	19 (26.4%)	-
Gender	Male	57	44 (77.2%)	13 (22.8%)	0.179
	Female	15	9 (60.0%)	6 (40.0%)	
BMI	Underweight	5	3 (60.0%)	2 (40.0%)	0.900
	Normal	16	13 (81.3%)	3 (18.8%)	
	Overweight	25	18 (72.0%)	7 (28.0%)	
	Obese	23	17 (73.9%)	6 (26.1%)	
	N/A	3	2 (66.7%)	1 (33.3%)	
Hgb	Normal(12-18)	47	36 (76.6%)	11 (23.4%)	0.661
	Abnormal	5	3 (60.0%)	2 (40.0%)	
	N/A	20	14 (70.0%)	6 (30.0%)	
Use of Medication	Yes	30	20 (66.7%)	10 (33.3%)	0.258
	None	42	33 (78.6%)	9 (21.4%)	
Chronic Disease	Yes	19	11 (57.9%)	8 (42.1%)	0.070
	No	53	42 (79.2%)	11 (20.8%)	
GI Symptoms	Yes	68	52 (76.5%)	16 (23.5%)	0.023
	No	4	1 (25.0%)	3 (75.0%)	
Constitutional Symptoms	Yes	6	4 (66.7%)	2 (33.3%)	0.687
	No	66	49 (74.2%)	17 (25.8%)	
Other	Yes	7	4 (57.1%)	3 (42.9%)	0.298
	No	65	49 (75.4%)	16 (24.6%)	

## Discussion

This study found some areas where FOBT confirmed the results of colonoscopy/sigmoidoscopy and histopathology, namely, in the distribution of positive/negative results and in the similar ratio of positive results found using FOBT and colonoscopy/sigmoidoscopy procedures. Though still lacking in the volume and persistence of observation, these findings support the thesis that FOBT has enough capability to be a reliable colorectal cancer screening mode. A Saudi Arabian study has claimed this potential, recognizing FOBT as a feasible screening method in the primary care environment. They based their proposition on the age-specific incidence rate (ASIR) of FOBT testing, which was found to be higher than the Saudi Cancer Registry figure, implying a high detection capability [10]. One study comparing FOBT with colonoscopy observed that FOBT sensitivity varied in different areas of malignancy. It appeared that FOBT accuracy benefits from programmed and repeated screening sessions [11].

More findings found disparities between FOBT and colonoscopy results, but not to a large degree that could outright negate the value of FOBT. Among patients with diagnosed acromegaly, FOBT found 18.8% (16 of 85 patients) positive and identified two patients with colonic adenocarcinoma and two with adenoma. Out of 29 patients, colonoscopy detected three (3.5%) cancers, 11 (12.9%) adenomas, and 15 (17.6%) hyperplastic polyps [12]. From the results of a Canadian study with about 5000 subjects, 346 FOBT patients were found positive, 41 (11.8%) of these had been confirmed to have colon cancer. Confirmed cancer findings from colonoscopy were 11 (5.6%) [13].

While clinical affirmation of FOBT to be at par with gold standard colonoscopy remains unestablished, several factors are still at play in the short- and long-term choice of colorectal cancer screening mode, as the following studies observed.

Among Saudi Arabians, stool FIT is the preferred screening method over FOBT or colonoscopy. This finding is seen as helping frame a screening program that conforms to population preferences. An information campaign is also recommended to increase willingness to undergo screening and promote early detection to avoid the burden and cost of late-stage cancer cases [14].

The role of screening in cancer management cannot be overstated. It is vital information in the evolution and advancement of the disease, as it impacts mortality as well as morbidity, which consequently impacts the burden of cost [15].

Another Saudi Arabian study identifies the invasive procedures of colonoscopy and pathology for their unappealing effect on the population. Favored screening methods were listed in order of preference: CT colonography (CTC), stool-based tests, colonoscopy, and flexible sigmoidoscopy. In a related study, a high-sensitivity guaiac fecal occult blood test (HSgFOBT) was preferred over scope-based methods [16].

Fierce physician recommendations may be a key factor in promoting the necessity and urgency of screening for colorectal cancer. A "doctor’s order" always provides that much-needed professional authority that is more likely to overcome barriers like personal preferences and fear of pain or discomfort from some procedures. This solution should necessarily be anchored on a greater understanding of the apparent and actual barriers that hamper optimal screening implementation [17].

Studies have advocated efficient screening operations and public education for awareness, and some have observed the positive impact of provider counseling via short, direct discussions about the screening the patient is about to undergo [17].

Limitation

The limitations of this study include the retrospective design, which cannot eliminate publication bias, and the subjective selection of data. The design is strengthened by compiling easily accessible data regarding a positive fecal occult blood test compared to colonoscopy and histopathology findings, which can help researchers analyze tabulated and pooled data for future studies. Also, the participants in this research are Saudi Arabian but they do not necessarily reflect the entire country's population. So, it's important to be cautious when extrapolating study results.

## Conclusions

While accuracy is a primary factor in choosing a colorectal cancer screening method, considerations like speed, convenience, and cost-effectiveness play into the ultimate objective of reducing mortality from the disease. FOBT was analyzed in this study, and the distribution pattern between positive and negative results had no significant statistical difference, suggesting the similarity of the two groups in their ratios of treatment outcomes. Among the variables identified, GI symptoms were found to have a significant statistical significance in predicting positive colonoscopy/sigmoidoscopy and histology. These findings, however, require repeated and long-term validation, necessitating comparable research in the future.
